# The reconstruction of 2,631 draft metagenome-assembled genomes from the global oceans

**DOI:** 10.1038/sdata.2017.203

**Published:** 2018-01-16

**Authors:** Benjamin J. Tully, Elaina D. Graham, John F. Heidelberg

**Affiliations:** 1 Center for Dark Energy Biosphere Investigations, University of Southern California, Los Angeles, CA 90089, USA; 2 Department of Biological Sciences, University of Southern California, Los Angeles, CA 90089, USA

**Keywords:** Genome, Metagenomics, Water microbiology, Bioinformatics

## Abstract

Microorganisms play a crucial role in mediating global biogeochemical cycles in the marine environment. By reconstructing the genomes of environmental organisms through metagenomics, researchers are able to study the metabolic potential of Bacteria and Archaea that are resistant to isolation in the laboratory. Utilizing the large metagenomic dataset generated from 234 samples collected during the *Tara* Oceans circumnavigation expedition, we were able to assemble 102 billion paired-end reads into 562 million contigs, which in turn were co-assembled and consolidated in to 7.2 million contigs ≥2 kb in length. Approximately 1 million of these contigs were binned to reconstruct draft genomes. In total, 2,631 draft genomes with an estimated completion of ≥50% were generated (1,491 draft genomes >70% complete; 603 genomes >90% complete). A majority of the draft genomes were manually assigned phylogeny based on sets of concatenated phylogenetic marker genes and/or 16S rRNA gene sequences. The draft genomes are now publically available for the research community at-large.

## Background & Summary

The global oceans are a vast environment in which many key biogeochemical cycles are performed by microorganisms, specifically the Bacteria and Archaea^1,2^. Assessing the role of individual microorganisms has been confounded due to limitations in growing and maintaining ‘wild’ organisms in the laboratory environment^3^. The advent of ‘-omic’ techniques, metagenomics, metatranscriptomics, metaproteomics, and metabolomics, has provided an avenue for exploring microbial diversity and function by skipping the necessity of culturing organisms, thus allowing researchers to study organisms for which growth conditions cannot be replicated. Specifically, the application of metagenomics, the sampling and sequencing of genetic material directly from environment, provides an avenue for reconstructing the genomic sequences of environmental Bacteria and Archaea^4–7^.

Through the *Tara* Oceans Expedition (2003–2010), thousands of samples were collected of marine life^8^, including more than 200 metagenomic samples targeting the viral and microbial components of the marine ecosystem from around the globe^9,10^. Several studies have started the process of reconstructing microbial genomes from these metagenomics samples, utilizing samples from the Mediterranean^11^ and the bacterial size fraction (0.2–3 μm)^12^. Here, we present >2,000 additional draft genomes from the Bacteria and Archaea estimated to be >50% complete reconstructed from 102 billion metagenomic sequences generated from multiple size fractions and depths at the 61 stations sampled during the *Tara* Oceans circumnavigation of the globe. Phylogenomic analysis suggests that this set of draft genomes includes highly sought after genomes that lack cultured representatives, such as: Group II (149) and Group III (12) Euryarchaeota, the Candidate Phyla Radiation (30), the SAR324 (18), the *Pelagibacteraceae* (32), and the *Marinimicrobia* (111).

We envision that these draft genomes will provide a resource for downstream analysis acting as references for metatranscriptomic^13^ and metaproteomic^14^ projects, providing the data necessary for large-scale comparative genomics within globally vital phylogenetic groups^15^, and allowing for the exploration of novel microbial metabolisms^16^. Non-redundant draft metagenome-assembled genomes have been deposited into the National Center for Biotechnology Information (NCBI) database and assembly data, including contigs used for binning, have been submitted to the public data repository figshare to allow for the further examination of metagenomic information that was not incorporated in to the draft genomes.

## Methods

These methods have been described in part previously^16^, but have now been applied to full dataset discussed below (Supplementary Fig. 1).

### Gathering metagenomics sequences & assembly

**Figure 1:**
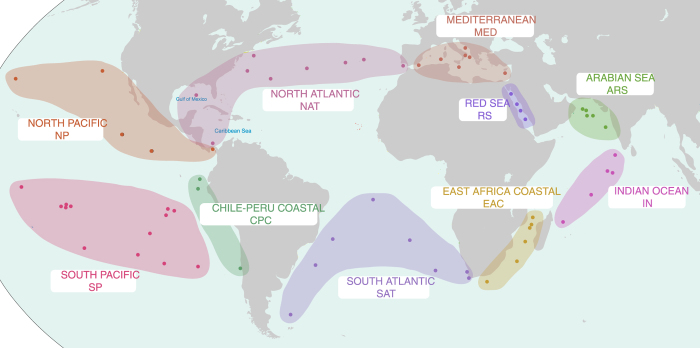
A map depicting the approximate locations of the *Tara* Oceans sampling stations from which metagenomics data was collected. Stations are grouped in to larger provinces based on Longhurst Provinces and site proximity. Province abbreviations are used for draft genome IDs. The map in Fig. 1 were modified under a CC BY-SA 3.0 license from ‘Oceans and Seas boundaries map’ by Pinpin.

An example of the methodology used to assemble the *Tara* Oceans metagenomes is available on Protocols.io (https://dx.doi.org/10.17504/protocols.io.hfqb3mw). All metagenomic sequences generated for 234 samples collected from 61 stations during the *Tara* Oceans expedition were accessed from the European Molecular Biology Laboratory-European Bioinformatics Institute (EMBL-EBI)^9,10^. Generally, samples were collected from multiple size fractions, commonly ‘viral’ (<0.22 μm), ‘girus’ (0.22–0.8 μm), ‘bacterial’ (0.22–1.6 μm), and ‘protistan’ (0.8–5.0 μm), at multiple depths, commonly at the surface (~5-m), deep chlorophyll maximum (DCM), and mesopelagic, from each station. Samples represent the filters from which DNA was extracted and sequenced (e.g., Station TARA007, girus filter fraction, surface depth), and multiple samples can belong to one station. The 61 stations were grouped in to 10 oceanic provinces as depicted in Fig. 1. Each sample was assembled individually using Megahit^17^ (v.1.0.3; parameters: --preset meta-sensitive). It should be noted that in several instances the size of samples from the South Pacific caused the Megahit assembly to fail; these samples were split to allow assembly and are noted in Table 1. Each of the 234 samples were assembled individually in an effort to avoid unresolvable assembly branches (commonly referred to as bubbles) caused by strain heterogeneity in closely related organisms. Strain heterogeneity from endemic organisms at different stations may cause breakages in the assembly, such that treating each sample individually increases the threshold at which organisms with limited strain heterogeneity may be successfully recovered. However, this assembly procedure does not resolve issues with abundant organisms with high degrees of strain heterogeneity within a single sample.

In total, over 102 billion paired-end reads were assembled into >562 million contigs (Table 1 (available online only); referred to as primary contigs). Primary contigs <2 kb in length were not used in downstream analysis. All primary contigs ≥2 kb in length from a province were processed using CD-HIT-EST^18^ (v4.6; parameter: -c 0.99) to reduce the computational load required for the secondary assembly by combining contigs with ≥99% semi-global identity. Primary contigs from the same oceanographic province were co-assembled using Minimus2^19^ (Fig. 1; AMOS v3.1.0; parameters: -D OVERLAP=100 MINID=95). Combining the Minimus2 generated contigs and the primary contigs that did not assemble with Minimus2, approximately 7.2 million contigs were generated for downstream analysis (Table 2; referred to as secondary contigs).

### Binning

An example of the methodology used to bin the *Tara* Oceans metagenomes is available on Protocols.io (https://dx.doi.org/10.17504/protocols.io.iwgcfbw). Metagenomic reads from each sample in a oceanic province were recruited against the set of secondary contigs generated from that same province using Bowtie2^20^ (v4.1.2; default parameters). Binning was performed using a custom BinSanity^21^ workflow. Coverage was determined using BinSanity-profile, which incorporates featureCounts^22^ to determine a reads·bp^−1^ coverage value for each contig from each sample. Coverage values were multiplied by 100 and log normalized (parameter: --transform scale). Then due to computational limitations imposed during the BinSanity binning method, the secondary contigs from each province were size selected (≥4–14 kb cutoffs) to choose approximately 100,000 contigs for binning (Table 2). Approximately 6 million secondary contigs remain un-binned and are available for analysis. Coverage values were only determined for contigs and samples from the same province to prevent instances where organisms with low abundance (or no abundance) values in different oceanic regions could lead to the convergence of unrelated contigs during the binning step and result in failure to resolve quality bins.

The binning using BinSanity was performed iteratively six times, with changes to the preference value after the first three iterations and a set parameter for iterations 4–6 in order to influence the degree of clustering (v0.2.5.5; parameters: -p [(1) −10, (2) −5, (3) −3, (4–6) −3] -m 4,000 -v 400 -d 0.95). Bins with high contamination (>10% contamination; see below) and low completion (<50% complete; see below) generated with BinSanity (using only coverage) were processed with the BinSanity-refinement script utilizing a set preference value (parameter: -p −25 -kmer 4). After the six iteration with BinSanity, bins with high contamination were processed two more times with BinSanity-refinement using variable preference values (parameter: -p [(6) −10, (7) −3]). After each BinSanity and BinSanity-refinement step, bins were assessed using CheckM^23^ (v1.0.3; parameters: lineage_wf) for completion and contamination estimates, which were used as cutoffs for inclusion in the final dataset (SupplementalTable1.xlsx, Data Citation 2). Bins were reassigned as a draft genome if: >90% complete with <10% contamination, 80–90% complete with <5% contamination, or 50–80% complete with <2% contamination. Bins that did not meet these criteria were combined for the next iteration of binning, except after the six iteration (see above). In total, 2,631 draft genomes were generated, with 1,491 of the genomes >70% complete, and 420 genomes meeting a high-quality threshold of >90% complete and <5% contamination (Supplementary Table 1). Genomes were provided identifiers with the format ***T**ara* **O**ceans **B**inned **G**enome (TOBG)—Province Abbreviation—Numeric ID (e.g., TOBG_NAT-221).

An additional 15,557 bins were generated containing at least five contigs that did not meet the criteria for reclassification as a draft genome. These bins may offer pertinent information for different downstream analyses. Bins of interest with high completion and high contamination can be manually assessed using tools, such as Anvi’o^24^, to generate a more accurate draft genome. For bins with <50% completion, it may be possible to combine two or more bins to generate a draft genome. And for bins with minimal or no phylogenetic markers assessment may reveal that they represent viral, episomal, or eukaryotic DNA sequences.

### Phylogenetic assignment

**Figure 2:**
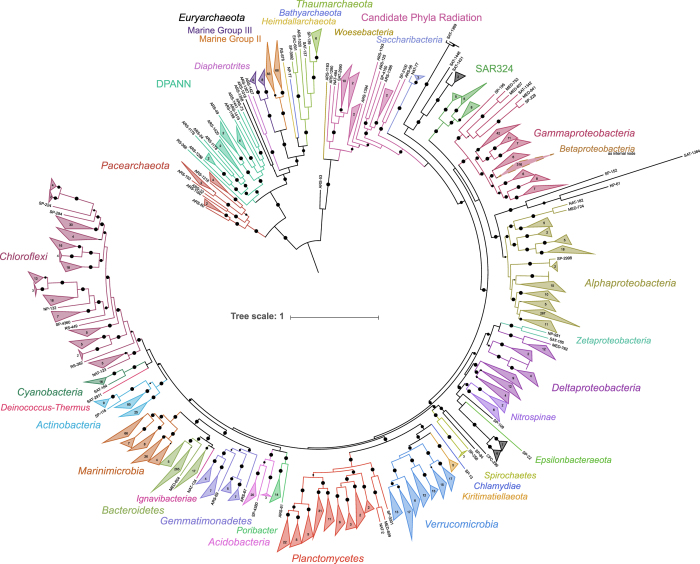
A maximum likelihood tree of the TOBG draft genomes based on 16 concatenated single-copy phylogenetic markers. Bootstrap values >0.75 are shown. Circle size representing the bootstrap value is scaled from 0.75–1.0. Nodes where the average branch length distance is <0.5 were collapsed and the number of draft genomes in each node are provided. The image was generated using the Interactive Tree of Life (iTOL; http://itol.embl.de/).

A multi-pronged approach was used to provide a phylogenetic assignment to all of the draft genomes. All of the secondary contigs had putative coding DNA sequences (CDSs) predicted using Prodigal^25^ (v2.6.2; -m -p meta). Contigs assigned to draft genomes and 7,041 complete and partial reference genomes (SupplementalTable2.xlsx, Data Citation 2) accessed from NCBI GenBank^26^ were searched for phylogenetic markers. Protein phylogenetic markers were detected using hidden Markov models (HMMs) collected from the Pfam database^27^ (Accessed March 2017) and identified using HMMER^28^ (v3.1b2; parameters: hmmsearch -E 1e-10). Two sets of single-copy markers recalcitrant to horizontal gene transfer were identified and used to construct phylogenetic trees; a set of 16 generally syntenic markers identified in Hug, *et al.*^29^ and an alternative set of 25 markers, for which 24 of the markers do not overlap in the Hug, *et al.* set (SupplementalTable3.xlsx, Data Citation 2). As the Hug, *et al.* marker set is syntenic, incomplete draft genomes may lack some or all of these markers. In order to accurately assign phylogeny to draft genomes without sufficient markers to be included with the Hug, *et al.* set, the alternative marker set consisted of additional single-copy phylogenetic markers^30^ present in a majority of the reference genomes. Draft and reference genomes were required to possess ≥10 and ≥15 markers for the Hug, *et al.* and alternative marker sets, respectively, to be included in downstream analysis. If multiple copies of the same marker were detected, neither copy was considered for further analysis. Each marker was aligned using MUSCLE^31^ (v3.8.31; parameter: -maxiters 8), trimmed using trimAL^32^ (v.1.2rev59; parameter: -automated1), and manually assessed. Alignments for each set of markers were concatenated. A maximum likelihood tree using the LGGAMMA model was generated using FastTree^33^ (v.2.1.10; parameters: -lg -gamma; SupplementalInformation1-HugTree.newick.txt, SupplementalInformation2-AltTree.newick.txt, Data Citation 2). Phylogenies were determined manually for 2,009 and 95 draft genomes for the Hug, *et al.* and alternative marker sets, respectively, based on the location of each draft genome on the respective trees (Supplementary Table 2). A simplified phylogenetic tree of the Hug, *et al.* phylogenetic marker set was constructed using the same parameters with only the alignments of the draft genomes for Fig. 2.

16S rRNA genes were predicted from draft genomes using RNAmmer^34^ (v1.2; parameters: -S bac -m ssu). 276 16S rRNA genes were detected and aligned using the SINA web portal aligner^35^ (https://www.arb-silva.de/aligner/). Aligned 16S rRNA gene sequences were added to the non-redundant 16S rRNA gene database (SSURef128 NR99) in ARB^36^ (v6.0.3) using the Parsimony (Quick) tool (default parameters). Each 16S rRNA gene sequence from a draft genome was assigned a putative phylogeny based on placement on the SSURef128 NR99 guide tree (Supplementary Table 2; SupplementalTable4.xlsx, Data Citation 2).

For the draft genomes, 81.3% were manually assigned a phylogeny based on the Hug, *et al.* marker set (2,009 draft genomes), the alternative marker set (95 draft genomes), or the 16S rRNA gene tree (35 draft genomes). The remaining 492 draft genomes were provided a putative phylogeny based on CheckM (Supplementary Table 2; SupplementalTable4.xlsx, Data Citation 2).

### Relative abundance

Several of the size fractions used to reconstruct bacterial and archaeal draft genomes were specifically designed to target different biological entities, such as double-stranded DNA viruses, giant viruses (giruses), and protists. In order to estimate the relative abundance of the draft genomes compared to only the total bacterial and archaeal community, a set of 100 previously identified HMMs for predominantly single-copy bacterial and archaeal markers^37,38^ were searched against the putative CDS of the secondary contigs from each province using HMMER (parameters: hmmsearch --cut_tc). From each province, the set of CDS identified by the marker HMMs could be used to approximate the total bacterial and archaeal community. Markers belonging to the draft genomes were identified. Based on the metagenomic reads recruited to the secondary contigs for each sample, the number of reads aligned to each marker in a sample was determined using BEDTools^39^ (v2.17.0; multicov default parameters). A length-normalized estimate of relative abundance for each draft genome in each sample in a province was determined using the following equation:

$$\frac{\sum \mathrm{Reads}\;{\mathrm{bp}}^{-1}\;\mathrm{TOBG}\;\mathrm{markers}}{\sum \mathrm{Reads}\;{\mathrm{bp}}^{-1}\;\mathrm{all}\;\mathrm{province}\;\mathrm{markers}}\times 100$$

**Figure 3:**
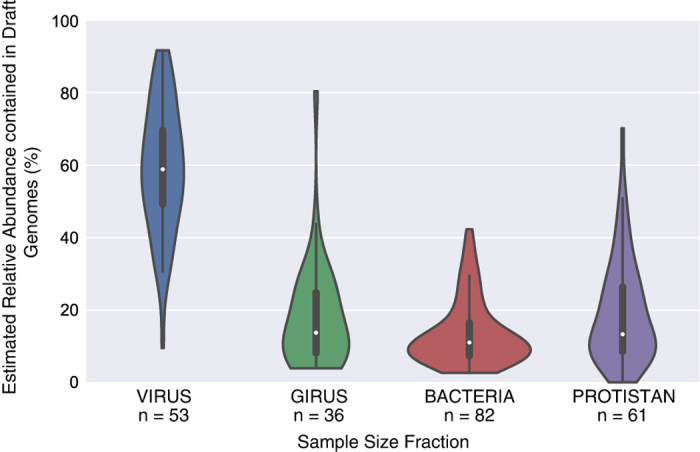


The relative abundance estimates of draft genomes indicate that the genomes generated for this study constitute only a small percentage of the total bacterial and archaeal abundance in each sample (Fig. 3; SupplementalTable5.xlsx, Data Citation 2). The draft genomes account for a higher percentage of the viral size fraction compared to other size fractions, accounting for ~60% of the total bacterial and archaeal community in that size fraction. This is likely due to the fact that the number of microbial organisms capable of passing through a 0.22 μm filter is limited and the overall microbial community in these samples is less complex, possibly resulting in increases in assembly efficiency and/or binning performance. On average, the draft genomes in the girus, bacterial, and protistan size fractions account for 14–19% of the total bacterial and archaeal communities. As such, the application of alternative binning methods to this same dataset should generate additional draft genomes^40^.

## Data Records

This project has been deposited at DDBJ/ENA/GenBank under the BioProject accession no. PRJNA391943 with the Whole Genome Shotgun project deposited under the accessions NYSJ00000000-NZZZ00000000 and PAAA00000000-PCDB00000000 (Data Citation 1). NCBI Assembly accession IDs for the 2,281 newly described draft genomes are listed in the ISA-Tab metadata record accompanying this Data Descriptor. Assembly sequence for the 324 genomes determined to be duplicates can be found in the TOBG-BINS.tar.gz files (Data Citation 2). Additional data is available through figshare, including copies of all draft genomes, all primary contigs, all secondary contigs, read count data for each secondary contig from each sample, and Supplementary Information and tables (Data Citation 2). The set of 100 HMMs for predominantly single-copy bacterial and archaeal markers from Albertsen, *et al.*^37^ is available on GitHub (https://github.com/MadsAlbertsen/multi-metagenome/blob/master/R.data.generation/essential.hmm).

## Technical Validation

Inclusion in this dataset requires that specific thresholds be achieved during the procedure discussed in the manuscript. Additional technical validation should be applied by researchers to confirm the accuracy of draft genomes used for specific downstream purposes.

## Usage Notes

The TOBG genomes have been generated using an automated process without manual assessment, as such, all downstream research should independently assess the accuracy of genes, contigs, and phylogenetic assignments for organisms of interest. Several of the draft genomes generated through this methodology appear to be identical, based on the Hug marker set phylogenomic tree, to genomes generated by Tully, *et al.*^11^ and Delmont, *et al.*^12^, these genomes have been identified (Supplementary Table 1) and in most cases duplicate genomes were not submitted to NCBI. In total, 186 draft genomes from this dataset, 68 from Tully, *et al.*^11^ and 118 from Delmont, *et al.*^12^, were determined to be identical to the previous work and not submitted to NCBI. However, draft genomes from this study that were estimated to be more complete than available through Delmont, *et al.*^12^ were submitted (*n*=198) to NCBI. In providing official nomenclature for submission to NCBI, priority was given to the Hug marker assignment, followed by the 16S rRNA assignment, then alternative marker assignment, and, finally, the CheckM assignment.

## Additional information

**How to cite this article:** Tully, B. J. *et al.* The reconstruction of 2,631 draft metagenome-assembled genomes from the global oceans. *Sci. Data* 5:170203 doi:10.1038/sdata.2017.203 (2018).

**Publisher’s note:** Springer Nature remains neutral with regard to jurisdictional claims in published maps and institutional affiliations.

## Supplementary Material



Supplementary Fig. 1

Supplementary Table 1

Supplementary Table 2

## Figures and Tables

**Table 1:** Statistics for the primary contigs generated for each of the 234 sample fractions (Table1_ReadsPrimaryContigs.xlsx, Data Citation 2)

**Site**	**Size fraction (girus, viral, bacteria, protistan)**	**Collection depth (surface, DCM, mesopelagic, epipelagic)**	**Province**	**No. of sequence reads**	**No. primary contigs**	**Total bp in assemblies (Mb)**	**N50 contig length (bp)**	**Longest contig (bp)**	**mean contig length (bp)**	**Recruitment rate to SECONARY contigs (%)**
TARA007	girus	DCM	Mediterranean	178,519,830	1,318,470	966	828	220,754	733	72.84
TARA007	girus	surface	Mediterranean	221,166,612	1,308,847	978	861	211,946	748	81.74
TARA007	protistan	DCM	Mediterranean	744,458,992	4,667,618	2,900	654	188,635	621	19.45
TARA007	protistan	surface	Mediterranean	265,432,098	2,590,120	1,418	564	18,444	548	25.58
TARA009	girus	DCM	Mediterranean	416,553,274	2,796,841	2,052	831	1,643,839	734	69.48
TARA009	girus	surface	Mediterranean	489,617,426	1,787,467	1,386	929	1,142,851	771	68.85
TARA009	protistan	DCM	Mediterranean	329,036,110	1,938,636	1,163	613	95,724	600	22.07
TARA009	protistan	surface	Mediterranean	370,813,078	1,700,350	1,006	588	292,050	592	22.53
TARA018	bacteria	DCM	Mediterranean	408,021,182	2,520,645	1,856	840	1,573,060	736	76.22
TARA018	bacteria	surface	Mediterranean	414,976,308	2,604,031	1,885	816	2,086,508	724	75.80
TARA023	bacteria	DCM	Mediterranean	147,400,552	1,273,576	925	830	213,456	727	76.08
TARA023	bacteria	surface	Mediterranean	149,566,010	1,237,617	892	825	134,179	721	75.98
TARA023	protistan	DCM	Mediterranean	508,610,652	2,707,801	1,845	734	336,689	682	28.23
TARA023	protistan	surface	Mediterranean	397,044,232	2,246,571	1,332	593	397,140	593	23.00
TARA025	bacteria	DCM	Mediterranean	386,627,816	2,516,865	1,809	806	388,546	719	69.77
TARA025	bacteria	surface	Mediterranean	457,560,422	2,326,838	1,722	857	330,773	740	75.57
TARA030	bacteria	DCM	Mediterranean	346,837,034	1,968,945	1,666	1,097	508,775	846	80.16
TARA030	bacteria	surface	Mediterranean	478,785,582	1,639,697	1,433	1,194	204,976	874	77.70
TARA030	protistan	DCM	Mediterranean	426,896,616	1,620,343	987	616	478,892	610	15.12
TARA030	protistan	surface	Mediterranean	430,029,974	1,838,588	1,136	628	287,782	618	22.36
TARA031	bacteria	surface	Red Sea	401,751,524	2,637,235	1,705	683	225,266	647	n.a.
TARA032	bacteria	DCM	Red Sea	394,022,740	2,425,270	1,723	781	428,757	711	n.a.
TARA032	bacteria	surface	Red Sea	397,670,070	2,362,538	1,509	668	344,626	639	n.a.
TARA033	bacteria	surface	Red Sea	397,670,070	2,362,538	1,073	770	240,965	699	n.a.
TARA034	bacteria	DCM	Red Sea	449,416,158	2,824,068	2,227	939	1,315,944	789	n.a.
TARA034	bacteria	surface	Red Sea	241,308,424	1,457,859	1,020	771	264,827	700	n.a.
TARA034	girus	surface	Red Sea	208,403,344	1,121,149	871	934	186,511	778	n.a.
TARA036	bacteria	DCM	Arabian Sea	402,101,650	2,377,674	1,612	733	223,432	678	54.56
TARA036	bacteria	surface	Arabian Sea	251,165,916	1,696,904	1,150	731	319,254	678	56.75
TARA036	girus	surface	Arabian Sea	47,924,602	350,701	245	774	72,154	699	62.34
TARA037	girus	mesopelagic	Arabian Sea	532,737,716	2,832,914	2,072	827	410,668	732	58.85
TARA037	protistan	mesopelagic	Arabian Sea	340,330,348	1,132,273	1,000	1,216	122,351	884	63.13
TARA038	girus	mesopelagic	Arabian Sea	157,533,556	934,763	685	822	661,953	734	54.27
TARA038	girus	surface	Arabian Sea	70,533,644	504,239	356	791	124,801	707	59.42
TARA038	protistan	DCM	Arabian Sea	328,205,338	1,812,989	1,071	605	100,905	591	20.41
TARA038	protistan	mesopelagic	Arabian Sea	321,388,280	1,932,086	1,516	943	459,954	785	49.48
TARA038	protistan	surface	Arabian Sea	375,209,692	2,389,728	1,365	570	231,924	571	17.79
TARA039	bacteria	DCM	Arabian Sea	338,044,892	2,333,110	1,617	754	236,017	693	50.42
TARA039	bacteria	mesopelagic	Arabian Sea	276,669,050	1,428,466	1,077	870	575,037	754	68.70
TARA039	girus	mesopelagic	Arabian Sea	183,842,664	1,139,408	903	954	609,350	793	58.33
TARA039	girus	surface	Arabian Sea	49,964,608	436,285	304	760	189,255	697	54.27
TARA041	virus	DCM	Indian Ocean	200,048,946	844,428	632	866	110,269	749	54.58
TARA041	virus	surface	Indian Ocean	85,317,778	619,858	461	867	309,423	744	58.85
TARA042	bacteria	DCM	Indian Ocean	430,039,794	2,382,038	1,669	767	212,579	701	46.59
TARA042	bacteria	surface	Indian Ocean	401,723,064	2,480,801	1,588	671	243,758	640	39.19
TARA042	virus	DCM	Indian Ocean	120,008,604	827,511	660	978	128,597	798	56.27
TARA042	virus	surface	Indian Ocean	99,112,476	586,326	470	1,025	388,068	803	67.43
TARA045	bacteria	surface	Indian Ocean	391,038,482	2,561,812	1,615	651	238,052	631	36.05
TARA046	girus	surface	Indian Ocean	119,610,982	645,882	509	964	898,395	788	65.93
TARA046	virus	surface	Indian Ocean	77,435,642	420,629	349	1,033	330,930	832	62.79
TARA056	bacteria	mesopelagic	East African Coastal Current	434,938,762	2,539,451	1,686	696	742,292	664	46.12
TARA056	bacteria	surface	East African Coastal Current	324,775,688	2,422,793	1,439	601	100,550	594	47.18
TARA056	virus	mesopelagic	East African Coastal Current	103,921,448	406,219	314	863	344,347	774	68.38
TARA056	virus	surface	East African Coastal Current	112,802,278	722,250	604	1,065	604,845	837	68.49
TARA057	bacteria	surface	East African Coastal Current	336,385,740	2,289,578	1,492	682	307,060	652	53.66
TARA058	bacteria	DCM	East African Coastal Current	337,711,862	2,632,119	1,715	680	307,060	652	52.16
TARA058	virus	DCM	East African Coastal Current	102,649,026	660,715	516	953	132,555	782	70.52
TARA062	bacteria	surface	East African Coastal Current	291,429,494	2,132,986	1,358	665	242,877	637	51.53
TARA062	virus	surface	East African Coastal Current	121,191,220	769,356	606	969	107,667	788	70.96
TARA064	bacteria	DCM	East African Coastal Current	410,378,996	2,608,625	1,659	666	269,266	636	47.41
TARA064	bacteria	mesopelagic	East African Coastal Current	244,932,320	1,767,190	1,075	625	65,774	608	30.69
TARA064	bacteria	surface	East African Coastal Current	629,462,328	4,394,496	2,691	630	108,622	612	52.51
TARA064	girus	DCM	East African Coastal Current	125,388,218	926,273	623	731	83,530	673	55.55
TARA064	protistan	mesopelagic	East African Coastal Current	390,795,372	47,800	29	756	32,104	625	1.65
TARA064	virus	DCM	East African Coastal Current	85,653,938	631,724	497	965	93,919	788	67.24
TARA064	virus	mesopelagic	East African Coastal Current	102,506,134	569,505	484	1,073	953,425	851	57.43
TARA064	virus	surface	East African Coastal Current	96,542,160	641,467	543	1,073	583,518	608	70.78
TARA065	bacteria	DCM	East African Coastal Current	433,566,456	2,543,340	1,498	596	158,682	589	49.98
TARA065	bacteria	mesopelagic	East African Coastal Current	446,725,782	3,107,471	1,861	610	303,918	599	32.22
TARA065	bacteria	surface	East African Coastal Current	290,200,094	1,909,554	1,216	664	113,492	637	51.51
TARA065	girus	DCM	East African Coastal Current	187,370,916	1,401,468	946	734	138,185	675	59.24
TARA065	girus	surface	East African Coastal Current	176,516,224	1,294,698	857	713	127,803	662	56.69
TARA065	virus	DCM	East African Coastal Current	113,406,914	784,229	648	1,033	498,271	827	67.64
TARA066	bacteria	DCM	East African Coastal Current	149,855,818	1,346,974	874	670	94,763	649	40.98
TARA066	bacteria	surface	East African Coastal Current	320,731,360	2,533,452	1,626	664	188,150	642	41.57
TARA066	virus	DCM	East African Coastal Current	93,801,200	555,878	474	1,095	891,763	853	66.58
TARA066	virus	surface	East African Coastal Current	87,897,252	516,881	460	1,181	898,395	892	67.55
TARA067	bacteria	surface	South Atlantic	157,314,750	1,347,634	1,007	879	237,021	748	51.48
TARA067	girus	surface	South Atlantic	697,082,396	2,910,380	2,383	1,036	449,174	819	61.69
TARA067	virus	surface	South Atlantic	879,440,068	412,456	377	1,356	898,395	914	68.73
TARA068	bacteria	DCM	South Atlantic	262,743,724	1,764,118	1,110	646	211,624	630	56.12
TARA068	bacteria	mesopelagic	South Atlantic	373,710,956	2,542,152	1,561	637	387,642	614	49.14
TARA068	bacteria	surface	South Atlantic	294,061,050	2,035,306	1,286	651	267,481	632	57.09
TARA068	girus	DCM	South Atlantic	627,763,012	3,746,847	2,580	748	323,424	689	68.40
TARA068	girus	mesopelagic	South Atlantic	362,537,996	2,654,130	1,761	710	154,842	664	51.26
TARA068	girus	surface	South Atlantic	97,121,250	630,411	2,911	760	225,875	695	72.83
TARA068	protistan	DCM	South Atlantic	695,701,562	4,187,552	1,280	514	186,625	525	50.10
TARA068	virus	mesopelagic	South Atlantic	89,633,440	509,267	489	897	666,812	777	56.74
TARA068	virus	surface	South Atlantic	449,406,884	2,439,771	477	1,342	745,358	938	76.08
TARA070	bacteria	mesopelagic	South Atlantic	389,679,635	1,749,423	1,168	709	533,014	668	39.19
TARA070	bacteria	surface	South Atlantic	262,754,638	1,675,586	1,038	640	387,645	620	64.79
TARA070	girus	mesopelagic	South Atlantic	722,583,172	2,938,950	1,987	716	683,357	676	66.42
TARA070	girus	surface	South Atlantic	82,514,066	292,815	3,579	752	473,356	694	64.65
TARA070	virus	mesopelagic	South Atlantic	742,578,192	5,160,189	281	1,381	945,611	961	75.60
TARA070	virus	surface	South Atlantic	94,440,814	601,127	540	1,218	605,778	900	82.56
TARA072	bacteria	DCM	South Atlantic	327,621,287	2,604,940	1,791	746	318,774	688	71.51
TARA072	bacteria	mesopelagic	South Atlantic	287,954,144	2,115,939	1,318	642	645,058	623	n.a.
TARA072	bacteria	surface	South Atlantic	420,077,668	2,965,977	1,864	652	148,227	629	47.84
TARA072	virus	DCM	South Atlantic	97,624,502	536,923	449	1,058	144,616	837	n.a.
TARA072	virus	mesopelagic	South Atlantic	193,526,010	1,104,923	844	905	456,824	764	n.a.
TARA072	virus	surface	South Atlantic	79,974,260	546,916	453	1,066	456,836	829	65.42
TARA076	bacteria	DCM	South Atlantic	433,352,388	2,577,859	1,687	699	298,119	655	n.a.
TARA076	bacteria	mesopelagic	South Atlantic	59,215,780	458,450	293	694	60,796	641	n.a.
TARA076	girus	DCM	South Atlantic	664,302,686	4,287,795	2,976	753	220,105	694	n.a.
TARA076	girus	mesopelagic	South Atlantic	391,269,294	2,358,816	1,680	782	443,072	713	n.a.
TARA076	girus	surface	South Atlantic	86,619,086	376,602	3,052	700	448,060	660	n.a.
TARA076	virus	DCM	South Atlantic	706,014,112	4,621,282	621	1,119	1,135,950	855	n.a.
TARA076	virus	mesopelagic	South Atlantic	117,740,296	727,275	335	782	443,072	713	n.a.
TARA076	virus	surface	South Atlantic	100,296,296	530,597	466	1,191	363,305	879	n.a.
TARA078	bacteria	DCM	South Atlantic	458,306,264	2,933,171	1,842	648	153,325	628	n.a.
TARA078	bacteria	mesopelagic	South Atlantic	484,317,850	3,077,729	1,915	647	391,597	622	n.a.
TARA078	girus	DCM	South Atlantic	717,300,708	4,816,605	3,369	768	362,325	700	n.a.
TARA078	girus	mesopelagic	South Atlantic	303,977,220	1,088,504	763	769	367,631	701	n.a.
TARA078	girus	surface	South Atlantic	609,624,124	4,339,400	2,833	693	284,920	653	n.a.
TARA078	virus	DCM	South Atlantic	76,302,668	469,799	584	1,119	395,306	866	n.a.
TARA078	virus	surface	South Atlantic	107,092,254	674,841	403	1,110	566,748	860	n.a.
TARA082	virus	DCM	South Atlantic	92,242,804	236,734	214	1,171	157,015	854	n.a.
TARA082	virus	surface	South Atlantic	83,754,456	251,672	197	1,088	457,670	835	n.a.
TARA093	bacteria	DCM	Chile-Peru Coastal Current	338,611,726	1,812,410	1,364	863	440,011	753	62.59
TARA093	bacteria	surface	Chile-Peru Coastal Current	274,983,484	1,842,493	1,359	833	433,729	738	61.11
TARA093	protistan	DCM	Chile-Peru Coastal Current	1,006,359,456	5,751,669	3,551	658	351,230	617	15.35
TARA093	protistan	surface	Chile-Peru Coastal Current	1,095,335,702	5,293,293	3,457	687	436,820	653	30.13
TARA102	protistan	DCM	Chile-Peru Coastal Current	1,379,777,772	7,528,562	4,567	645	186,352	607	21.61
TARA102	protistan	mesopelagic	Chile-Peru Coastal Current	326,198,334	1,914,049	1,006	512	369,681	526	13.29
TARA102	protistan	surface	Chile-Peru Coastal Current	1,253,380,792	5,915,885	3,735	689	108,426	631	28.65
TARA102	virus	DCM	Chile-Peru Coastal Current	83,338,338	482,593	428	1,235	457,912	888	63.19
TARA102	virus	mesopelagic	Chile-Peru Coastal Current	102,166,560	632,611	506	1,003	457,656	800	52.07
TARA102	virus	surface	Chile-Peru Coastal Current	82,514,022	398,171	339	1,165	536,583	853	72.65
TARA109	protistan	DCM	Chile-Peru Coastal Current	1,103,449,668	6,493,345	3,899	633	105,788	601	19.13
TARA109	protistan	mesopelagic	Chile-Peru Coastal Current	746,296,748	3,462,161	1,832	531	311,927	529	21.60
TARA109	protistan	surface	Chile-Peru Coastal Current	1,131,293,272	6,806,574	4,184	657	455,077	615	21.90
TARA109	virus	DCM	South Pacific	164,307,434	705,315	530	879	110,332	752	64.73
TARA109	virus	surface	South Pacific	91,637,852	644,379	556	1,133	457,719	863	60.76
TARA094	bacteria	surface	South Pacific	460,018,862	2,354,210	1,900	989	439,038	807	50.38
TARA096	bacteria	surface	South Pacific	377,820,080	2,197,395	1,488	733	284,592	677	44.52
TARA096	protistan	DCM	South Pacific	411,445,340	2,243,098	1,222	534	183,113	545	18.14
TARA096	protistan	surface	South Pacific	401,619,602	2,069,652	1,168	560	134,007	565	11.43
TARA098	bacteria	DCM	South Pacific	255,245,468	2,146,916	1,373	670	160,328	640	42.80
TARA098	bacteria	mesopelagic	South Pacific	450,447,948	3,780,623	2,335	639	195,288	618	32.50
TARA098	bacteria	surface	South Pacific	253,142,740	1,799,731	1,160	683	141,561	645	42.71
TARA098	protistan	DCM	South Pacific	395,142,260	1,927,462	1,016	520	126,378	528	44.58
TARA099	bacteria	surface	South Pacific	338,549,582	2,379,626	1,626	738	281,788	684	45.56
TARA100	protistan	DCM-total	South Pacific	1,216,104,648	5,657,514	n.a.	n.a.	n.a.	n.a.	14.96
TARA100	protistan	DCM-a	South Pacific	363,691,834	1,418,334	827	585	223,453	584	n.a.
TARA100	protistan	DCM-b	South Pacific	493,756,292	2,832,506	1,595	581	14,162	563	n.a.
TARA100	protistan	DCM-c	South Pacific	358,656,522	1,406,674	793	564	389,248	564	n.a.
TARA100	protistan	mesopelagic	South Pacific	351,098,942	1,647,164	954	576	321,593	580	43.99
TARA100	protistan	surface	South Pacific	1,326,576,228	6,694,120	3,892	595	190,392	581	10.35
TARA100	virus	DCM	South Pacific	99,958,986	420,580	370	1,218	453,359	882	69.41
TARA100	virus	mesopelagic	South Pacific	83,920,264	566,025	442	964	701,104	783	38.80
TARA100	virus	surface	South Pacific	93,781,526	302,956	248	1,077	628,013	820	75.32
TARA110	bacteria	DCM	South Pacific	423,500,782	3,393,442	2,248	698	367,057	662	54.09
TARA110	bacteria	mesopelagic	South Pacific	385,005,800	2,866,452	1,849	678	207,768	645	43.22
TARA110	bacteria	surface	South Pacific	321,797,088	2,753,073	1,749	657	397,901	635	54.03
TARA110	protistan	DCM-total	South Pacific	753,695,370	3,605,143	n.a.	n.a.	n.a.	n.a.	18.93
TARA110	protistan	DCM-a	South Pacific	368,784,520	2,023,556	1,062	530	31,710	525	n.a.
TARA110	protistan	DCM-b	South Pacific	384,910,850	1,581,587	851	534	174,660	538	n.a.
TARA110	protistan	mesopelagic	South Pacific	357,179,962	2,138,971	1,200	552	382,759	561	20.29
TARA110	protistan	surface-total	South Pacific	1,153,271,554	6,963,165	n.a.	n.a.	n.a.	n.a.	18.60
TARA110	protistan	surface-a	South Pacific	353,771,502	3,304,465	1,969	635	13,413	596	n.a.
TARA110	protistan	surface-b	South Pacific	450,624,510	2,228,194	1,223	556	158,640	549	n.a.
TARA110	protistan	surface-c	South Pacific	348,875,542	1,430,506	786	547	268,238	550	n.a.
TARA111	bacteria	mesopelagic	South Pacific	417,176,234	2,744,418	1,754	677	207,790	639	39.32
TARA111	protistan	DCM-total	South Pacific	765,733,752	4,039,885	n.a.	n.a.	n.a.	n.a.	19.65
TARA111	protistan	DCM-a	South Pacific	372,817,728	2,083,224	1,199	594	19,223	576	n.a.
TARA111	protistan	DCM-b	South Pacific	392,916,024	1,956,661	1,101	578	17,964	563	n.a.
TARA111	protistan	mesopelagic	South Pacific	424,975,474	603,165	480	976	431,613	796	12.59
TARA111	protistan	surface-total	South Pacific	801,686,136	3,935,566	n.a.	n.a.	n.a.	n.a.	17.34
TARA111	protistan	surface-a	South Pacific	362,293,856	2,179,012	1,307	632	15,718	600	n.a.
TARA111	protistan	surface-b	South Pacific	439,392,280	1,756,554	905	507	94,481	515	n.a.
TARA111	virus	DCM	South Pacific	102,845,312	356,328	340	1,427	550,760	956	74.77
TARA111	virus	surface	South Pacific	95,232,394	336,420	267	1,058	105,049	796	74.70
TARA112	bacteria	DCM	South Pacific	412,959,348	3,096,304	1,838	695	572,156	626	
TARA112	bacteria	mesopelagic	South Pacific	303,174,866	2,935,274	2,029	695	334,869	655	39.92
TARA112	bacteria	surface	South Pacific	399,008,374	2,898,952	1,837	674	1,031,167	634	42.02
TARA122	girus	DCM	South Pacific	831,226,140	3,386,282	2,909	1,080	569,971	859	67.82
TARA122	girus	mesopelagic	South Pacific	736,940,856	4,068,270	2,698	700	566,739	663	51.38
TARA122	girus	surface	South Pacific	737,598,522	2,652,604	2,165	980	1,368,461	816	67.16
TARA122	protistan	DCM	South Pacific	649,370,158	4,310,143	2,225	499	278,972	516	17.04
TARA122	protistan	mesopelagic	South Pacific	571,394,218	75,488	28	371	12,941	372	0.28
TARA122	protistan	surface-total	South Pacific	1,293,949,316	7,824,957	n.a.	n.a.	n.a.	n.a.	17.14
TARA122	protistan	surface-a	South Pacific	689,964,424	4,511,903	2,512	568	107,287	557	n.a.
TARA122	protistan	surface-b	South Pacific	603,984,892	3,313,054	1,669	488	158,529	504	n.a.
TARA122	virus	DCM	South Pacific	94,581,636	342,218	341	1,502	237,809	998	85.07
TARA122	virus	mesopelagic	South Pacific	126,487,568	833,691	659	903	706,445	791	54.46
TARA122	virus	surface	South Pacific	120,037,536	414,804	396	1,320	712,941	957	81.82
TARA123	girus	epipelagic	South Pacific	858,470,910	4,380,259	3,227	836	735,770	737	67.29
TARA123	girus	surface	South Pacific	749,554,388	2,488,778	2,064	987	842,147	829	73.22
TARA123	protistan	epipelagic	South Pacific	576,095,390	2,856,566	1,598	552	279,371	560	30.49
TARA123	protistan	surface	South Pacific	768,591,240	3,672,985	2,086	576	880,243	568	12.52
TARA123	virus	epipelagic	South Pacific	117,495,592	449,817	457	1,575	182,476	1,018	80.77
TARA123	virus	surface	South Pacific	103,788,030	402,512	388	1,394	582,726	965	80.87
TARA124	girus	epipelagic	South Pacific	822,925,152	3,935,725	3,261	1,013	906,923	829	74.85
TARA124	girus	surface	South Pacific	804,362,170	2,914,023	2,472	1,044	996,906	848	71.70
TARA124	protistan	epipelagic	South Pacific	1,230,860,846	5,170,334	3,003	570	651,229	581	24.72
TARA124	protistan	surface	South Pacific	2,193,739,450	9,113,992	5,771	667	897,170	633	23.07
TARA124	virus	epipelagic	South Pacific	76,666,526	370,994	362	1,423	205,055	976	85.18
TARA124	virus	surface	South Pacific	133,841,438	461,626	440	1,324	745,367	954	82.92
TARA125	girus	epipelagic	South Pacific	956,324,374	2,956,725	2,637	1,130	568,375	892	72.87
TARA125	girus	surface	South Pacific	903,597,880	3,427,442	3,303	1,376	1,413,687	964	71.23
TARA125	protistan	epipelagic	South Pacific	579,239,460	3,480,777	1,954	554	463,473	561	28.52
TARA125	protistan	surface	South Pacific	2,182,494,756	9,156,739	5,380	606	483,302	588	11.86
TARA125	virus	epipelagic	South Pacific	126,300,944	352,568	349	1,496	512,338	992	28.52
TARA125	virus	surface	South Pacific	111,598,866	319,501	314	1,455	193,719	985	86.10
TARA128	bacteria	DCM	South Pacific	297,815,870	2,164,606	15,045	749	1,091,607	695	58.05
TARA128	bacteria	surface	South Pacific	306,384,522	2,152,096	1,417	690	325,354	658	57.17
TARA128	protistan	DCM	South Pacific	1,228,121,166	5,922,143	3,573	636	1,060,229	603	13.23
TARA128	protistan	surface	South Pacific	1,308,863,638	6,804,942	4,090	633	1,368,388	601	15.96
TARA133	bacteria	DCM	North Pacific	359,284,260	2,738,902	2,036	848	421,301	744	48.12
TARA133	bacteria	mesopelagic	North Pacific	437,816,192	2,663,466	1,660	654	527,331	623	36.17
TARA133	bacteria	surface	North Pacific	539,113,764	3,414,508	2,570	867	235,669	753	49.39
TARA137	bacteria	DCM	North Pacific	385,071,042	2,069,078	1,553	858	348,433	751	58.99
TARA137	bacteria	surface	North Pacific	371,142,378	2,827,258	1,827	673	206,837	646	40.99
TARA137	protistan	DCM	North Pacific	897,564,856	4,550,412	2,669	601	605,967	587	20.38
TARA137	protistan	mesopelagic	North Pacific	384,153,332	2,596,725	1,779	743	428,415	685	49.07
TARA137	protistan	surface	North Pacific	1,242,625,464	7,405,806	4,836	720	152,543	653	18.43
TARA137	virus	DCM	North Pacific	72,196,088	510,284	399	971	110,093	783	44.66
TARA137	virus	mesopelagic	North Pacific	65,674,652	445,263	362	955	743,871	813	42.16
TARA137	virus	surface	North Pacific	72,756,492	469,660	375	982	187,663	801	58.64
TARA138	protistan	DCM	North Pacific	984,440,322	5,494,456	3,232	607	328,704	588	16.16
TARA138	protistan	mesopelagic	North Pacific	599,026,030	3,177,295	2,172	746	520,054	684	40.80
TARA138	protistan	surface	North Pacific	360,832,782	2,327,739	1,292	549	1,091,714	555	15.35
TARA138	virus	surface	North Pacific	86,921,234	476,972	368	925	98,412	773	56.94
TARA004	bacteria	DCM	North Atlantic	476,293,096	3,230,937	2,234	753	2,086,245	692	61.51
TARA004	bacteria	surface	North Atlantic	404,336,892	2,599,459	1,835	781	1,859,026	706	54.04
TARA141	bacteria	surface	North Atlantic	342,398,030	2,381,702	1,687	789	1,184,498	709	48.31
TARA142	bacteria	DCM	North Atlantic	311,581,824	2,332,926	1,662	790	476,594	713	53.10
TARA142	bacteria	mesopelagic	North Atlantic	328,237,008	2,476,759	1,582	665	241,588	639	39.67
TARA142	bacteria	surface	North Atlantic	314,283,624	2,300,282	1,650	796	659,598	718	54.39
TARA145	bacteria	mesopelagic	North Atlantic	354,224,626	2,635,068	1,739	704	604,226	660	34.57
TARA145	bacteria	surface	North Atlantic	352,030,928	2,481,128	1,766	787	270,268	712	48.91
TARA146	bacteria	mesopelagic	North Atlantic	307,846,576	2,764,796	1,629	602	490,727	589	32.20
TARA146	bacteria	surface	North Atlantic	338,943,134	2,772,579	1,919	752	339,109	692	54.45
TARA146	protistan	mesopelagic	North Atlantic	388,319,946	2,027,090	1,277	648	1,292,170	630	28.25
TARA146	protistan	surface	North Atlantic	340,205,542	2,053,519	1,252	656	24,270	610	20.83
TARA148	protistan	surface	North Atlantic	1,078,181,620	5,989,279	3,908	699	2,698,294	653	23.05
TARA149	protistan	mesopelagic	North Atlantic	632,883,922	2,447,499	1,445	606	502,600	590	25.54
TARA149	protistan	surface	North Atlantic	520,481,158	3,574,647	2,238	651	116,237	626	22.44
TARA150	bacteria	DCM	North Atlantic	364,054,734	3,081,379	2,146	758	486,854	696	56.13
TARA150	protistan	DCM	North Atlantic	775,883,764	4,767,635	2,903	635	421,300	609	22.67
TARA150	protistan	surface	North Atlantic	1,025,677,076	5,688,556	3,233	580	83,644	568	17.05
TARA151	bacteria	DCM	North Atlantic	369,538,288	3,277,737	2,238	737	298,340	683	50.18
TARA151	protistan	DCM	North Atlantic	431,037,388	2,783,258	1,590	577	223,537	571	18.12
TARA152	bacteria	mesopelagic	North Atlantic	345,574,560	2,948,541	1,846	645	463,812	626	34.53
TARA152	bacteria	mixed	North Atlantic	388,462,874	3,070,311	2,046	713	201,743	667	52.09
TARA152	bacteria	surface	North Atlantic	329,240,054	2,508,617	1,704	733	200,712	679	55.08
TARA152	protistan	DCM	North Atlantic	1,762,378,578	9,544,963	5,843	647	450,904	612	17.77
TARA152	protistan	mesopelagic	North Atlantic	345,740,494	2,364,548	1,406	594	807,748	595	24.01
SUM				102,321,613,478	562,600,489	384,383	803 (mean)	432,318 (mean)	700 (mean)	48.06 (mean)

**Table 2:** Statistics for each province on the number secondary contigs generated, the number of contigs binned and corresponding length cutoff, and the number of draft genomes reconstructed.

**Province**	**No. of Secondary Contigs**	**Size Cutoff (kb)**	**No. of Binned Contigs**	**No. of Draft Genomes**
Mediterranean	660,937	7.5	95,506	360
Red Sea	328,325	5.0	84,936	180
Arabian Sea	525,636	6.0	99,649	194
Indian Monsoon	285,238	4.0	93,760	72
East Africa Coastal Current	613,778	7.0	91,053	208
South Atlantic	1,373,173	11.5	96,972	360
Chile Peru Coastal	857,548	5.5	95,557	146
South Pacific	807,193	14.0	104,598	536
North Pacific	943,809	7.0	96,396	254
North Atlantic	804,316	8.5	104,848	321
SUM	7,199,953	-	963,275	2,631
