# Supplementary figures and images for: The reconstruction of 2,631 draft metagenome-assembled genomes from the global oceans
Source: Sci Data. 2018 Jan 16;5:170203. doi: 10.1038/sdata.2017.203 (PMC5769542; doi:10.1038/sdata.2017.203)

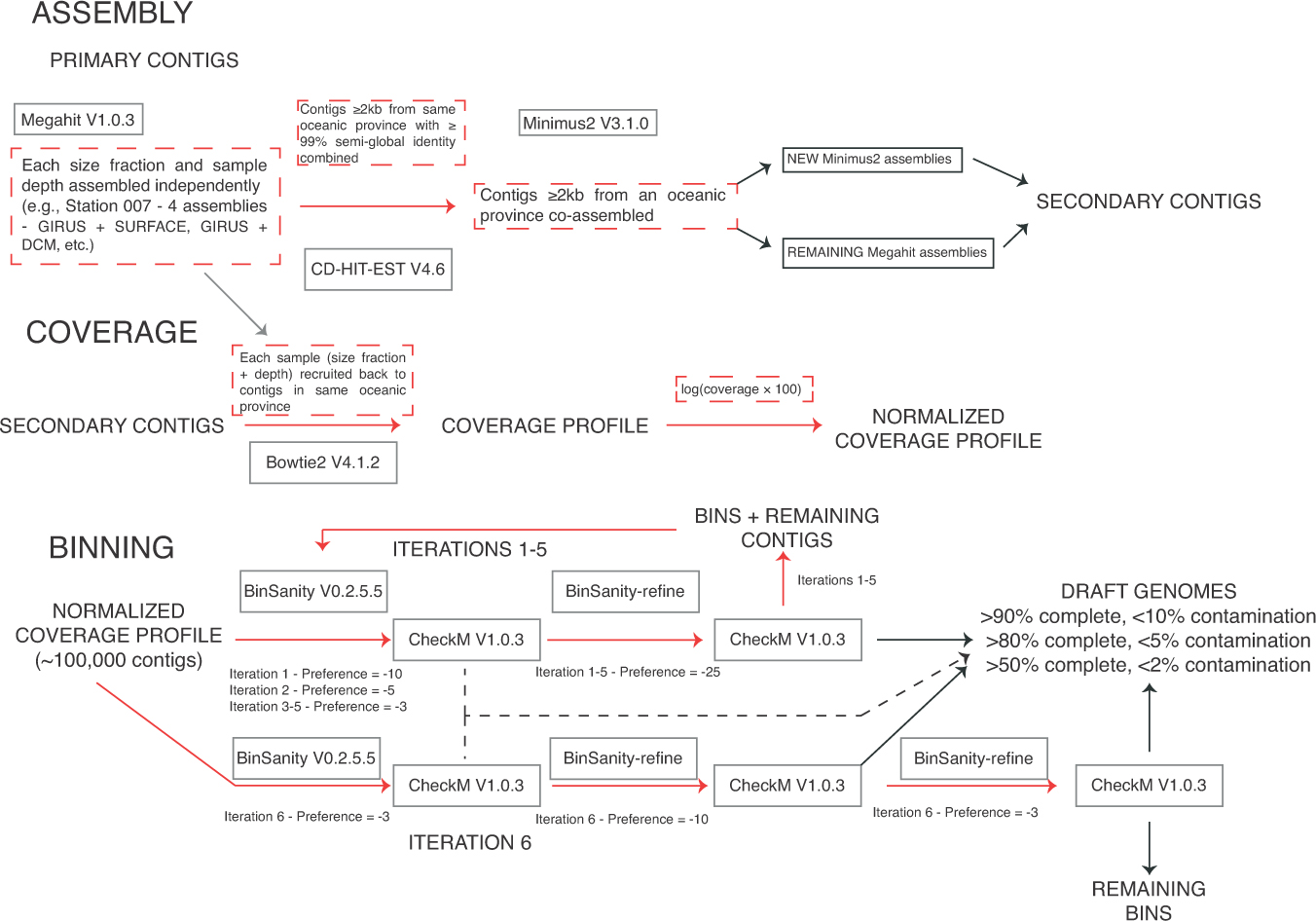

Supplement: Supplementary Fig. 1 [file sdata2017203-s2.jpg]
